# Punicalagin Induces Serum Low-Density Lipoprotein Influx to Macrophages

**DOI:** 10.1155/2016/7124251

**Published:** 2016-07-19

**Authors:** Dana Atrahimovich, Soliman Khatib, Shifra Sela, Jacob Vaya, Abraham O. Samson

**Affiliations:** ^1^Department of Oxidative Stress and Human Diseases, MIGAL–Galilee Research Institute, 11016 Kiryat Shmona, Israel; ^2^Tel-Hai College, 12208 Upper Galilee, Israel; ^3^Faculty of Medicine in the Galilee, Bar-Ilan University, 1311502 Safed, Israel; ^4^Eliachar Research Laboratory, Western Galilee Hospital, 22100 Nahariya, Israel

## Abstract

High levels of circulating low-density lipoprotein (LDL) are a primary initiating event in the development of atherosclerosis. Recently, the antiatherogenic effect of polyphenols has been shown to be exerted via a mechanism unrelated to their antioxidant capacity and to stem from their interaction with specific intracellular or plasma proteins. In this study, we investigated the interaction of the main polyphenol in pomegranate, punicalagin, with apolipoprotein B-100 (ApoB100) that surrounds LDL. Punicalagin bound to ApoB100 at low concentrations (0.25–4 *μ*M). Upon binding, it induced LDL influx to macrophages in a concentration-dependent manner, up to 2.5-fold. In contrast, another polyphenol which binds to ApoB100, glabridin, did not affect LDL influx. We further showed that LDL influx occurs specifically through the LDL receptor, with LDL then accumulating in the cell cytoplasm. Taken together with the findings of Aviram et al., 2000, that pomegranate juice and punicalagin induce plasma LDL removal and inhibit macrophage cholesterol synthesis and accumulation, our results suggest that, upon binding, punicalagin stimulates LDL influx to macrophages, thus reducing circulating cholesterol levels.

## 1. Introduction

Low-density lipoprotein (LDL) particles are the major peripheral tissues providing cholesterol in the human circulation, and they play a key role in the development of atherosclerosis [1]. LDL is surrounded by a single copy of apolipoprotein B-100 (ApoB100) [2] which binds the LDL receptor on the cell surface of target tissues [1].

Punicalagin is a soluble polyphenol isolated from pomegranate with potent antioxidative properties. Punicalagin protects macrophage cells from lipid accumulation and foam cell formation [3–5]. Similarly, coadministration of punicalagin with statin significantly protects against macrophage foam cell formation and inhibits macrophage cholesterol biosynthesis. The use of statins in combination with pomegranate juice in hypercholesterolemic patients enables lowering the dosage of the former, thereby preventing its side effects, such as increases in liver enzymes, muscle problems, cognitive loss, neuropathy, pancreatic and hepatic dysfunction, and sexual dysfunction [6, 7]. Pomegranate juice supplementation to atherosclerotic mice reduced macrophage lipid peroxidation, cellular cholesterol accumulation, and development of atherosclerosis [5]. The antiatherogenic effect of punicalagin is known to stem from its antioxidant capacity [8]. However, antioxidant activity cannot be the sole explanation for polyphenols' cellular effects* in vivo* since they are poorly absorbed through the gut into the bloodstream and extensively metabolized in the small intestine, liver, and colon; thus, their bioavailability is often poor [9, 10].

Another antiatherogenic polyphenol is glabridin, isolated from licorice root. Its antiatherogenic properties are assumed to derive from its strong antioxidant capacity [11]. Recently, we showed that, apart from it being an antioxidant, glabridin can protect plasma protein through specific binding [12]. In fact, alongside our research, accumulating evidence in the literature suggests that polyphenols interact directly with enzymes, membranes, receptors, and cell or plasma proteins and modulate the activity of key proteins involved in cell signaling [13–16]. Polyphenols' beneficial effect might thus be exerted via a mechanism that is not necessarily related to their antioxidant capacity.

Cells acquire cholesterol through uptake of lipoproteins and through* de novo* synthesis. Yet (with the exception of steroidogenic tissues), they are unable to catabolize it. Since excess unesterified cholesterol is toxic to cells, organisms have developed several ways to protect themselves from cholesterol accumulation [17]. Macrophages are the best example of this “self-protection”; they take up dead cells containing a large amount of cholesterol, modified lipoproteins, and other extracellular debris. Macrophages take up more cholesterol per cell than any other cell type and protect themselves from cholesterol toxicity by two pathways: one is the esterification of cholesterol to cholesteryl ester. However, accumulation of high levels of cholesteryl ester may lead to the formation of foam cells and, later, to atherogenesis. The second and major line of defense against cholesterol toxicity is high-density lipoprotein (HDL) cholesterol efflux. In addition, in comparison to other cells, macrophages have additional pathways of cholesterol efflux [17]. Excess “peripheral” cholesterol is returned to the liver where the whole-body steady-state level of cholesterol is maintained.

In this study, a possible interaction of punicalagin with ApoB100 and the biological consequences of this interaction were investigated. It was shown that punicalagin binds specifically to ApoB100 and that upon binding it induces LDL influx to macrophages via the LDL receptor; on the other hand, glabridin, which also binds to ApoB100, did not affect LDL influx. These results provide a new mechanism—different from the classical mechanism of “antioxidant activity”—by which punicalagin reduces cholesterol levels in the circulation and attenuates atherosclerosis.

## 2. Materials and Methods

### 2.1. J774A.1 Macrophage Cell Line

J774A.1 murine macrophage cells were purchased from the American Tissue Culture Collection (ATCC, Rockville, MD). The cells were grown at 37°C, 5% CO_2_ in Dulbecco's Modified Eagle's Medium (DMEM) containing glucose (4500 mg/L), 2 mM glutamine, 10% w/v fetal calf serum (FCS), 1% w/v pyruvate, and 0.5% w/v penicillin, streptomycin, and nystatin (all chemicals purchased from Sigma-Aldrich).

### 2.2. Human LDL Isolation

LDL was prepared from human plasma taken from fasting normolipidemic volunteers (approved for research by Helsinki Committee regulations). It was separated from the plasma by discontinuous density gradient ultracentrifugation [18] and dialyzed against saline with disodium ethylenediaminetetraacetate (EDTA) (1 mM, pH 7.4). LDL was diluted in phosphate buffered saline (PBS) to 1 mg protein/mL and dialyzed twice, for 1 h each time, and once more overnight against PBS at 4°C to remove EDTA (PBS and EDTA were purchased from Sigma-Aldrich).

### 2.3. LDL Oxidation

LDL (100 mg protein/L) was incubated with 10 *μ*mol CuSO_4_/L (Sigma-Aldrich) under gentle shaking for 3 h at 37°C. The formation of conjugated dienes was monitored by measuring the increase in absorbance at 234 nm. Measurements were carried out using a SpectraMax M2 Reader [8].

### 2.4. Fluorescence-Quenching Measurements

Measurements were performed using a previously reported procedure [19]. Briefly, the solution was prepared in a 96-well black enzyme-linked immunosorbent assay (ELISA) plate (Greiner Bio-One, Germany). To each well, 2 *μ*L polyphenol [glabridin, catechin, or quercetin (in ethanol) or punicalagin in double distilled water (DDW)] was added to 5 *μ*L LDL or apolipoprotein B-100 (ApoB100) diluted in PBS buffer, to give a final polyphenol concentration in the range of 0.25 to 4 *μ*M and a final LDL or ApoB100 concentration of 25 or 10 *μ*g protein/mL, respectively. ApoB100 was purchased from Abcam, USA, quercetin and catechin were purchased from Sigma-Aldrich, glabridin was isolated from licorice root extract [11], and punicalagin was a generous gift from Professor Michael Aviram of the Lipid Research Laboratory, Faculty of Medicine, Technion-Israel Institute of Technology, Haifa.

Fluorescence emission intensity was measured within 30 min of adding the polyphenol to the LDL or ApoB100 solution (25 or 37°C, resp.).

Fluorescence measurements were performed with an Infinite M200 PRO fluorescence spectrophotometer (Tecan) with emission spectra recorded from 320 to 450 nm at an excitation wavelength of 290 nm. If needed, the inner filter effect, which can decrease the fluorescence intensity, was corrected by using the following relationship:

(1) $$\begin{array}{c} F_{corr}=F_{obs}\times e^{\left(A_{ex}+A_{em}\right)/2}=F_{obs}\times e^{\left(\varepsilon_{ex}CL+\varepsilon_{em}CL\right)/2}, \end{array}$$

where *F*_corr_ and *F*_obs_ are the corrected and observed fluorescence intensities, respectively, *ε*_ex_ is 0.0165 *μ*M^−1^ cm^−1^ and *ε*_em_ is 0.0034 *μ*M^−1^ cm^−1^, and *L* is the well path length [20, 21]. Fluorescence quenching can occur via two different major mechanisms: static and dynamic. Both quenching pathways are described by the Stern-Volmer equation:

(2) $$\begin{array}{c} \frac{F_{0}}{F}=1+K_{sv}\left[\;Q\;\right], \end{array}$$

where *F*_0_ and *F* are the fluorescence intensities in the absence and presence of quencher, respectively, *K*_sv_ is the Stern-Volmer quenching constant, and [*Q*] is the quencher concentration. For dynamic quenching, *K*_sv_ can be written as *K*_*q*_ *τ*_0_:

(3) $$\begin{array}{c} K_{sv}=K_{q}\tau_{0}, \end{array}$$

where *K*_*q*_ is the quenching rate constant of the bimolecule and *τ*_0_ is the lifetime of the fluorophore in the absence of quencher, which is approximately 10^−8^ s for a Trp residue [22]. Binding parameters were calculated as described previously [12]. For static quenching, the equilibrium between free and bound molecules can be described by

(4) $$\begin{array}{c} log\left(\;\frac{F_{0}-F}{F}\;\right)=\log K_{a}+n\log\left[\;Q\;\right], \end{array}$$

where *K*_*a*_ is the binding constant, reflecting the degree of interaction between ApoB100/LDL and the polyphenols, and *n* is the number of binding sites specifying the number of polyphenol molecules bound to the macromolecule. Thermodynamic parameters were calculated as described previously [12]. To characterize the ApoB100-punicalagin interaction, the thermodynamic parameters enthalpy (Δ*H*), entropy (Δ*S*), and free energy (Δ*G*) were calculated. Δ*H* can be estimated indirectly by examining the temperature dependence of *K*_*a*_ and using (5). Δ*G* was estimated from (6) based on the binding constants at different temperatures, and Δ*S* was estimated from their relationship (see (7)):

(5) lnKa2Ka1=1T1−1T2ΔHR,

(6) ΔG=−RTln⁡Ka,

(7) ΔG=ΔH−TΔS,

where *K*_*a*1_ and *K*_*a*2_ are the binding constants at temperatures *T*_1_ and *T*_2_, respectively, and *R* is the gas constant.

### 2.5. LDL Influx by J774A.1 Macrophages

LDL (1 mg protein/mL) was incubated with 10 *μ*g/mL fluoroisothiocyanate (FITC) (purchased from Pierce, USA) for 1 h at room temperature in the dark and then dialyzed twice, for 1 h each time, and once more overnight against carbonate buffer (pH 9) at 4°C to remove excess FITC. FITC-conjugated LDL (LDL-FITC) was used for cellular uptake studies. J774A.1 macrophages were incubated at 37°C for 3 h with LDL-FITC at a final concentration of 25 *μ*g protein/mL in DMEM enriched with 20% (w/v) BSA instead of FCS. LDL uptake was determined by flow cytometry [23]. Measurements of cellular fluorescence were determined by fluorescence-activated cell sorting (FACS) (FACSCalibur 4CA) at 510–540 nm after excitation at 488 nm with an argon ion laser. To determine the effect of the polyphenol (glabridin, catechin, quercetin, or punicalagin) on LDL influx, LDL-FITC was incubated with the polyphenol for 15 min before adding it to DMEM for 3 h.

To confirm that the influx occurs through the LDL receptor, macrophages were incubated simultaneously with LDL-FITC (25 *μ*g protein/mL) and unlabeled LDL (12, 25, or 50 *μ*g protein/mL) to create competitive inhibition.

### 2.6. Confocal Microscopy Analysis

Macrophages were incubated at 37°C for 3 h with LDL-FITC at a final concentration of 25 *μ*g protein/mL in the presence or absence of 2 *μ*M punicalagin and observed using a Zeiss LSM 700 confocal laser scanning microscope at 63x magnification. A vertical stack through the *z*-axis of the cells was created with the 488 nm laser and images were collected at 1 *μ*m intervals. Axio Observer.Z1 was used to process the images.

### 2.7. Statistical Analysis

Statistical analysis was carried out using GraphPad Prism 5.01. Student's paired *t*-test was used to compare the means of two groups. Each experiment was repeated separately at least three times and was always performed in triplicate. Results are presented as mean fluorescence intensity (MFI) with significance determined at *p* < 0.01 (*∗*) or *p* < 0.001 (*∗∗*).

## 3. Results

### 3.1. Tryptophan- (Trp-) Fluorescence Quenching

**Scheme 1:**
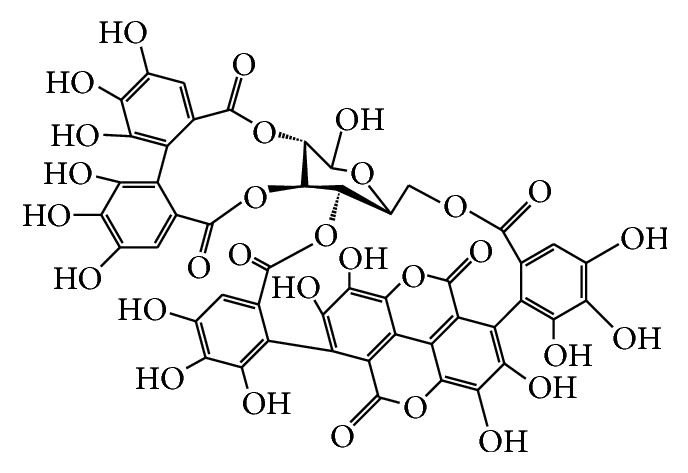
Punicalagin.

**Figure 1:**
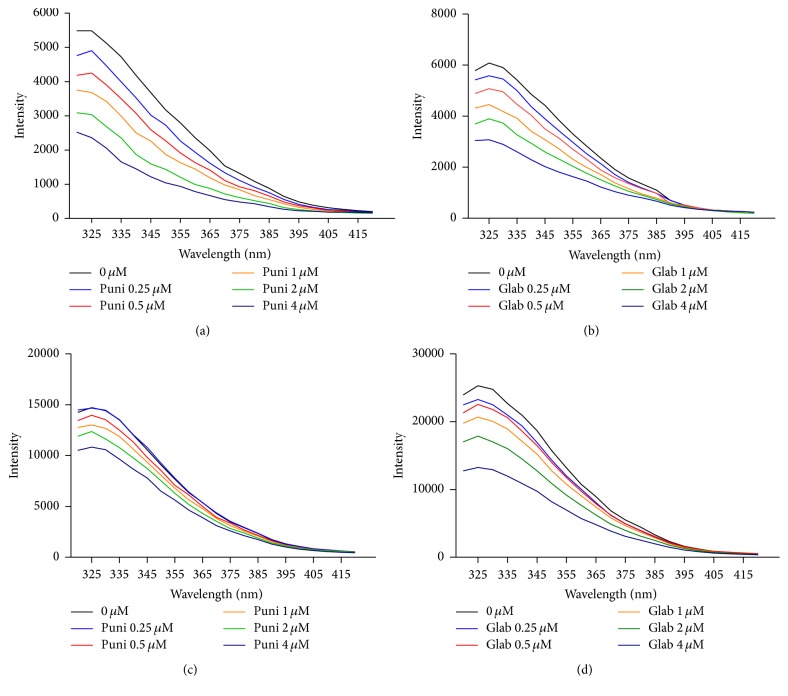
Fluorescence spectra of ApoB100 and LDL in the presence of punicalagin (puni) or glabridin (glab) at various concentrations. Fluorescence spectra of ApoB100 in the presence of various concentrations of punicalagin (a) and glabridin (b) and of LDL in the presence of various concentrations of punicalagin (c) and glabridin (d). In all solutions, the total concentration of ApoB100 and LDL was 0.01 and 0.025 mg/mL, respectively, and the polyphenol concentration was 0.25, 0.5, 1, 2, and 4 *μ*M (*λ*_ex_ = 290 nm, pH 7.4, and *T* = 298 K). Each experiment was repeated separately at least three times and was always performed in triplicate.

**Figure 2:**
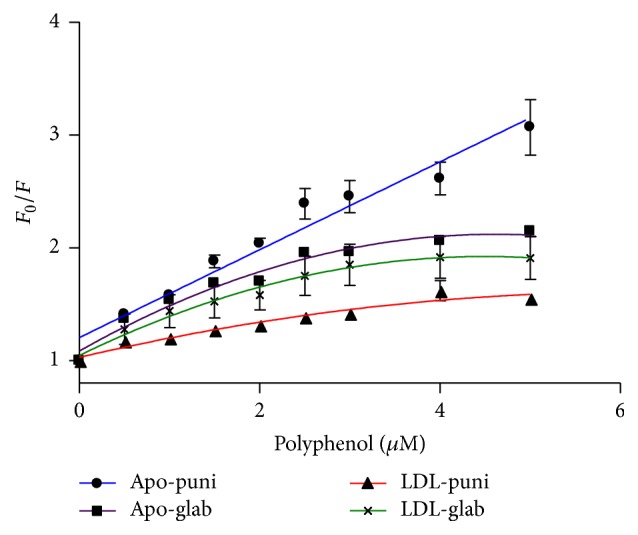
Stern-Volmer plots of the fluorescence quenching of ApoB100 and LDL by glabridin (glab) and punicalagin (puni). Each experiment was repeated separately at least three times. Results are presented as mean ± SD. *R* ^2^ = 0.9 and *p* < 0.0001 for the linear plot (ApoB100-punicalagin interaction).

Punicalagin (Scheme 1) and glabridin were assayed for possible binding with the LDL particle and its ApoB100 protein.  Figure 1 shows the emission spectra of ApoB100 in the presence of various concentrations of punicalagin (Figure 1(a)) and glabridin (Figure 1(b)) and of LDL in the presence of various concentrations of punicalagin (Figure 1(c)) and glabridin (Figure 1(d)) in the 320–415 nm range with excitation at 290 nm (*T* = 298 K, pH 7). Both glabridin and punicalagin quenched the Trp-fluorescence of ApoB100 and LDL in a concentration-dependent manner. Other polyphenol antioxidants that were examined, such as catechin and quercetin, did not quench the Trp-fluorescence of ApoB100 or LDL (data not shown). The Stern-Volmer curve (*F*_0_/*F* versus polyphenol concentration), shown in Figure 2, was only linear for the interaction of punicalagin with ApoB100 at the tested concentrations, indicating static or dynamic single-type quenching [24].

Quenching constant (*K*_*q*_) of ApoB100 initiated by punicalagin was calculated, using (3), to be 3.895 × 10^13^ M^−1^ s^−1^, which is much greater than the maximum diffusion collision quenching rate constant of various drugs with proteins (2 × 10^10^ M^−1^ s^−1^). This indicated that ApoB100 quenching by punicalagin is not initiated by dynamic collision but via stable complex formation [21, 24]. The ApoB100-glabridin interactions and the interactions with LDL particles, however, were not stable but diffusion dependent and binding parameters of the interaction cannot be determined (Figure 2).

### 3.2. Binding Constant and Binding Sites

Static quenching was demonstrated for the interaction of ApoB100 with punicalagin by the fact that the Stern-Volmer plot did not show any significant deviation from linearity toward the *y*- or *x*-axis at the reported punicalagin concentrations (Figure 2) and by the quenching constant (*K*_*q*_) value (see (3), Section 2). These results suggest a specific interaction between ApoB100 and punicalagin and that the binding parameters can be determined. However, the quenching of ApoB100 and LDL fluorescence by glabridin involves both static and dynamic quenching, as demonstrated by the fact that the Stern-Volmer plot deviates from linearity toward the *x*-axis, which indicates some site inaccessibility [20]. Thus, for glabridin, binding parameters of the interaction cannot be determined.

For static quenching and complex formation the binding parameters between punicalagin and ApoB100 can be determined using (4), Section 2. A plot of log(*F*_0_ − *F*)/*F* versus log⁡[*Q*], where *Q* is the polyphenol concentration, was used to determine the binding constant (*K*_*a*_), 3.78 × 10^6^ M^−1^, and the number of binding sites (*n*) which was close to 1 and not significantly affected by pH or temperature. These values indicate a single binding site for punicalagin in ApoB100 with a high affinity interaction (Table 1).

### 3.3. Thermodynamic Parameters and Nature of the Binding Forces

Thermodynamic parameters and nature of the binding forces were calculated for the interaction between punicalagin and ApoB100 using (5), (6), and (7), in Section 2. Table 1 shows negative values for Δ*G* and positive values for Δ*H* and Δ*S*. Such thermodynamic results indicate that the interaction is spontaneous and mainly entropy-driven [21].

### 3.4. LDL Influx to J774A.1 Macrophages

**Figure 3:**
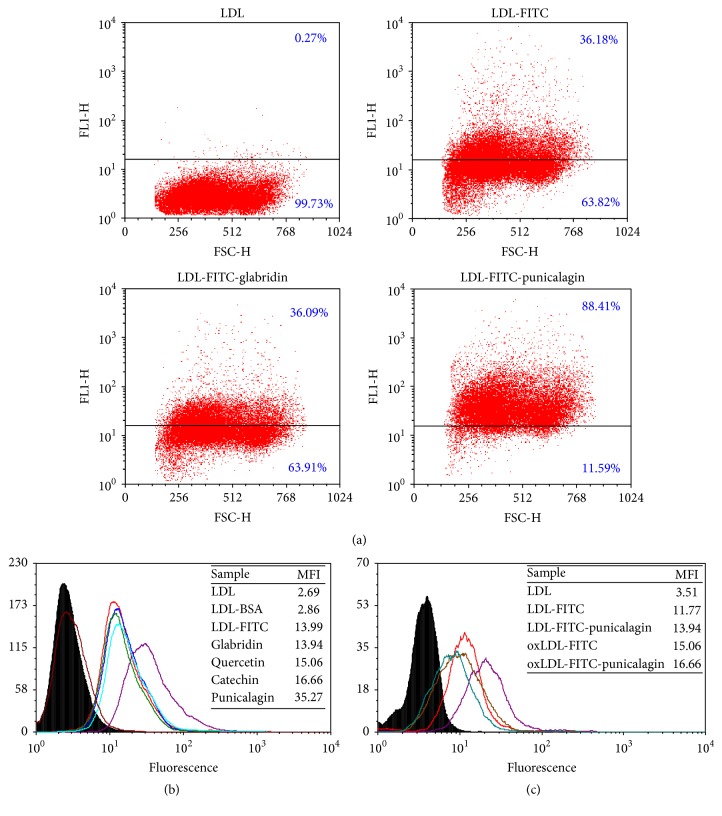
LDL or oxLDL influx into macrophages. (a) The ability of glabridin or punicalagin (2 *μ*M) to induce LDL influx to macrophages. (b) FACS histogram of macrophages incubated with LDL-FITC upon addition of 2 *μ*M punicalagin (purple), glabridin (blue), quercetin (green), or catechin (light-blue). Note that only punicalagin affects LDL influx as displayed by the curve shift compared to the control curves (red, brown, and black) representing macrophages incubated with LDL-FITC (positive control), LDL (negative control), and FITC-conjugated BSA (negative control), respectively. (c) FACS histogram of macrophages incubated with LDL-FITC (red) upon addition of 2 *μ*M punicalagin (purple) or oxLDL-FITC (brown) upon addition of 2 *μ*M punicalagin (green). LDL cellular fluorescence was measured in mean fluorescence intensity (MFI) or percent fluorescent cells.

**Figure 4:**
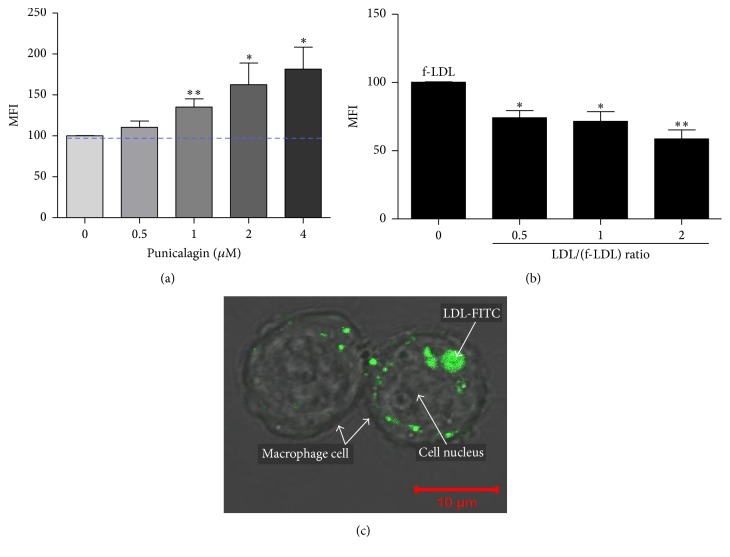
Punicalagin induces LDL influx. (a) Dose-response effect of 0.5–4 *μ*M punicalagin on LDL influx. (b) Competitive macrophage influx upon adding LDL and LDL-FITC (f-LDL) at various concentrations simultaneously. (c) LDL-FITC particles, upon punicalagin incubation, accumulate in the cell cytoplasm around the nucleus. Each experiment was repeated separately at least three times. LDL cellular fluorescence was measured in MFI with significance determined at *p* < 0.01 (*∗*) or *p* < 0.001 (*∗∗*).

The effect of various polyphenols on LDL influx into macrophages is shown in Figure 3.  Figure 3(a) shows that, upon macrophage incubation with 2 *μ*M punicalagin, LDL-FITC influx (cell fluorescence intensity) increased from 36% to 88%. On the other hand, the same concentration of glabridin had no effect on LDL influx (from 36.18% to 36.09%). Interestingly, only punicalagin (in purple) affected LDL influx, as displayed by the curve shift compared to the control curves (in red, black, and brown). No shift was observed for the blue, green, and light-blue curves, representing glabridin, quercetin, and catechin, respectively. Remarkably, LDL influx into the macrophage increased by up to 2.5-fold, mean fluorescence intensity (MFI) from 13 to 35 (Figure 3(b)), only in those cells that were incubated with 2 *μ*M punicalagin but not in the cells incubated with glabridin. This finding was unexpected, since both glabridin and punicalagin bind the LDL particle at the same concentrations. It should be noted that, under incubation of oxidized LDL (oxLDL) with macrophages, influx to macrophages is not affected by punicalagin (MFI values were 15.06 and 16.66 in the absence and presence of 2 *μ*M punicalagin, resp.). This is because oxLDL penetrates the macrophages through a different receptor, termed “scavenger” receptor (Figure 3(c)). The ability of punicalagin to increase LDL influx to macrophages was concentration-dependent.  Figure 4(a) demonstrates that while 2 *μ*M punicalagin bound to LDL increased cell MFI by 60%, 4 *μ*M punicalagin increased the LDL influx by 80%.

Next, we attempted to determine whether LDL influx occurs specifically through the LDL receptor. First, the cells were simultaneously incubated with LDL and LDL-FITC in various ratios to create competition. When cells were incubated with LDL (12 *μ*g protein/mL) + LDL-FITC (25 *μ*g protein/mL), macrophage MFI decreased by 30%; when cells were incubated with an LDL concentration that was twice that of LDL-FITC, MFI decreased by ≈45% (Figure 4(b)). Finally, images of a vertical *z* stack of two macrophage cells that were treated with LDL-FITC upon 2 *μ*M punicalagin incubation were taken. This image confirms that LDL particles indeed penetrate and accumulate in the cell cytoplasm. In Figure 4(c), a central image of the *z* stack shows LDL-FITC particles accumulated in the cell cytoplasm around the nucleus. It should be noted that, under incubation of oxidized LDL (oxLDL) with macrophages, influx to macrophages is not affected by punicalagin (MFI values were 15.06 and 16.66 in the absence and presence of 2 *μ*M punicalagin, resp.). This is because oxLDL penetrates the macrophages through a different receptor, termed “scavenger” receptor.

## 4. Discussion

Dietary polyphenols are found in fruit, vegetables, nuts, and teas [25]. The benefits of consuming polyphenols are commonly assumed to stem from their antioxidant activity, which may contribute to preventing diseases such as cancer, cardiovascular disease, and neurodegenerative disorders [26, 27]. In favor of its antioxidant capacity, punicalagin is antiatherogenic. However, much uncertainty surrounds its mechanism of action as punicalagin concentration in the blood hardly reaches the level needed for effective antioxidant activity (10–100 *μ*M) [15]. Recent studies suggest that the cellular effects of polyphenols are mediated by their interaction with specific intracellular or plasma proteins [28]. For example, punicalagin interacts with BSA [14]. The present study aimed to investigate a possible interaction of punicalagin with ApoB100 and the biological consequences of such an interaction.

Trp-fluorescence technique has been widely applied to the study of protein-drug interactions, as changes in the emission spectra of Trp can be seen in response to ligand binding or denaturation [24]. ApoB100 (and LDL) has 37 Trp residues [29] and natural fluorescence quenching can be used to measure its binding affinities. Both glabridin and punicalagin bound LDL or ApoB100 (with no shift in *λ*_em_), while catechin and quercetin did not (Figure 1).

The type of interaction between ApoB100 and punicalagin was interpreted from their fluorescence-quenching spectra [24] and was found to be the only strong and stable one. The values obtained for *n* indicated a single binding site for punicalagin in ApoB100 (Table 1). Thermodynamic parameters indicated that hydrophobic forces play a major role in the punicalagin-ApoB100 interaction [21].

Macrophages are central to the initiation and progression of atherosclerosis and can be highly appropriate targets for therapy. To examine the biological consequences of polyphenol's interaction with the LDL particle, macrophage cells were incubated with LDL-FITC solution after the latter had been incubated with each polyphenol, to examine the effect of each polyphenol on LDL influx. Similarly, as a negative control, cells were incubated first with the polyphenol without any LDL, washed to remove free excess polyphenol, and then incubated with LDL-FITC solution to examine a possible effect of the polyphenol alone on the cell (and in particular on cell LDL receptor). No effects on cells or LDL influx were observed. Supplementary Figure 1 (in Supplementary Material available online at http://dx.doi.org/10.1155/2016/7124251) shows that, upon incubation of the studied cells with LDL/LDL-FITC for 3 hours, no foam cell formation was evident. The cells did not change their morphology, even after incubation with LDL/LDL-FITC for 16 hours. We may conclude that, under the present experimental conditions, the interaction of punicalagin with LDL leads specifically to LDL influx to the macrophages without their conversion into foam cells.

This result highlighted the specificity of the consequences of such interaction between polyphenol and protein (upon macrophages incubation with 2 *μ*M punicalagin, LDL influx increased up to 2.5-fold while the same concentration of glabridin did not affect LDL influx (Figure 3(b))) and that punicalagin induction of LDL influx is in a concentration-dependent manner (Figure 3(c)).

Macrophages are phagocytes that engulf cellular debris and pathogens. We were interested in corroborating the concept that LDL influx occurs specifically through LDL receptor and that macrophages do not take up these particles nonspecifically by endocytosis as part of their defensive activity. FITC reagent was bound to BSA (for which LDL has no known receptor) in the same procedure as LDL-FITC to show that, upon incubation with punicalagin, there is no BSA influx into the cells (Figure 3(b)). In another experiment, competition for LDL receptor was generated by incubating the macrophages with LDL and LDL-FITC simultaneously. Adding LDL in a 1/1 ratio with LDL-FITC led to a 30% reduction in LDL-FITC influx. Increasing the ratio of LDL to LDL-FITC to 2/1 led to a 45% reduction in LDL-FITC influx (Figure 4(b)). These results validate the assumption that LDL influx occurs through the LDL receptor.

We postulate that punicalagin binds to ApoB100 in close proximity to the LDL receptor-binding site. Upon binding, punicalagin changes the protein's conformation and might increase LDL's affinity for LDL receptor. Similarly, the conformation of ApoB100 on the surface of the LDL particle is likely to depend on the composition of the core lipids, the surface phospholipid content, and the diameter of the LDL particle [30]. Thus, punicalagin probably interacts with both the lipid part of the LDL particle and the protein, which induces LDL influx into the macrophage. Finally, a vertical *z* stack of macrophage cells confirmed LDL penetration and accumulation in the cells (Figure 4(c)).

As LDL influx into hepatic cells may contribute to fatty liver disease [31], LDL absorption into hepatic cells in the presence or absence of 2 *μ*M punicalagin was also examined (data not shown). Unlike macrophage cells, LDL influx into hepatic cells (hepG2) was not affected by punicalagin when cells were exposed to similar LDL concentrations.

It is important to note that punicalagin from ingestion of pomegranate juice or extract does not reach high concentrations in the blood [15]. It is largely metabolized to ellagic acid through hydrolysis in the small intestine and over time by the gut bacteria to circulating urolithins [32]. Therefore, therapeutic administration of punicalagin preferentially should not be oral but rather intravenous. The results presented in this paper are collected from* in vitro* experiments. We are now examining the* in vivo* effect of punicalagin, using subcutaneously implanted osmotic mini-pumps. Serum lipoprotein parameters of mice will be determined after 28 days' exposure to punicalagin.

This study shows that punicalagin binds to a hydrophobic site of ApoB100 and to LDL, which may change the conformation of LDL's bound protein, ApoB100, and enhance its affinity for LDL receptor. LDL influx is induced and cholesterol accumulates in the macrophage cell without foam cell formation. In a future study, the effect of punicalagin on HDL's ability to remove excess cholesterol from these cells to the liver will be explored to determine the mechanism by which punicalagin lowers cholesterol blood concentration as reported in the literature [4, 5, 8] and attenuates the development of atherosclerosis.

## Supplementary Material

The first symptom of atherosclerosis disease is the process of oxidized LDL take-up by macrophage through a different receptor than the LDL is taken-up. Upon this process, cells are become what is called "foam cell". Although the LDL particles used for the experiment were not oxidized (as was determined by measuring the level of lipid peroxidation products on the LDL particles), we, nevertheless, aimed to make sure that LDL influx by the cells does not lead to Foam cell formation. Cells were incubated for 16 h with LDL/LDL-FITC (although the experiment took only 3 h), watched under a microscope and compared to macrophages incubated for 16 h with oxidized LDL. Morphologically, foam cells have distorted shape, and they are full of oxidized lipid as can be seen in Supplementary Fig. 1C. Supplementary Fig. 1A and B shows that upon LDL/LDL-FITC incubation the cells looked the same as in the control (cells that weren't incubated with LDL/LDL-FITC), what means that no foam cell formation observed upon the LDL/LDL-FITC influx.

## Figures and Tables

**Table 1:** Binding constant (*K*_*a*_), number of binding sites (*n*), and thermodynamic parameters for the ApoB100-punicalagin interaction (see Scheme 1).

*T* (K)	*K*_*a*_ (1/M)	*n*	Δ*H* (kJ/mol)	Δ*G* (kJ/mol)	Δ*S* (J/mol K)
310	3.78 · 10^6^	0.95 ± 0.04	51.2	−39.05	291
298	1.7 · 10^6^	0.7 ± 0.05		−35.54	
