# Supplementary material for: Punicalagin Induces Serum Low-Density Lipoprotein Influx to Macrophages
Source: Oxid Med Cell Longev. 2016 Jul 19;2016:7124251. doi: 10.1155/2016/7124251 (PMC4969581; doi:10.1155/2016/7124251)

Punicalagin induces serum low-density lipoprotein (LDL) influx to

macrophages

Dana Atrahimovich^1, 2^, Soliman Khatib^1^, Shifra Sela^2, 3^, Jacob Vaya^1^, and Abraham O. Samson^2*^

**Supplementary Figure 1.**


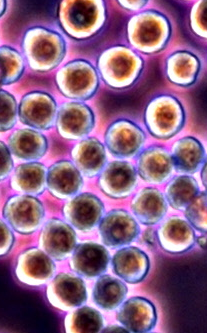

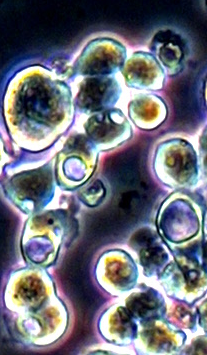

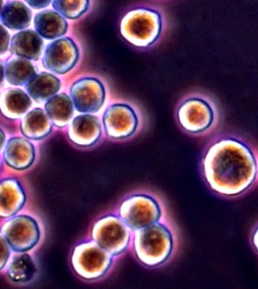


A[Type a quote from the document or the summary of an interesting point. You can position the text box anywhere in the document. Use the Drawing Tools tab to change the formatting of the pull quote text box.]

B

C

**Supplementary Figure 1. LDL influx to macrophages does not form foam cells**. Fig. 1 shows the cells before (A) and after (B) 16 h incubation of macrophage cells with LDL/LDL-FITC. No foam cell formation or morphological changes can be observed. Macrophage cells upon 16 h incubation with oxLDL/oxLDL-FITC are shown in (C). Foam cell formation characterized by cell distortion following mass of oxidized lipid absorption can be noticed.

**Table of Contents/Abstract Graphics**

**Punicalagin induces serum low-density lipoprotein (LDL) influx to macrophages**

Dana Atrahimovich^1, 2^, Soliman Khatib^1^, Jacob Vaya^1^ and Abraham O. Samson ^2*^


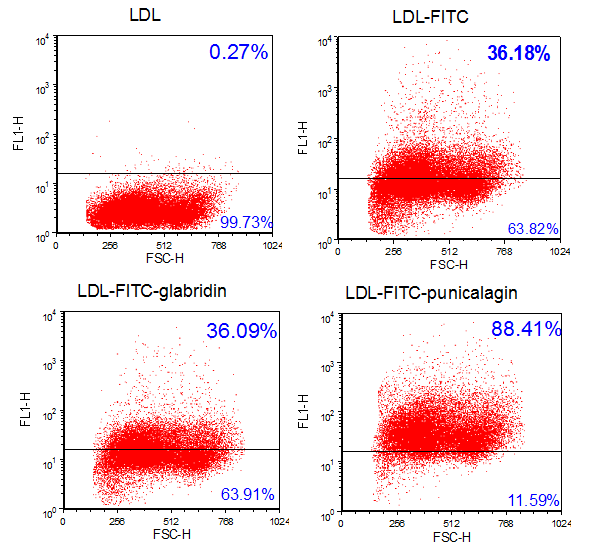

Supplement: Supplementary file 1 — The first symptom of atherosclerosis disease is the process of oxidized LDL take-up by macrophage through a different receptor than the LDL is taken-up. Upon this process, cells are become what is called "foam cell". Although the LDL particles used for the experiment were not oxidized (as was determined by measuring the level of lipid peroxidation products on the LDL particles), we, nevertheless, aimed to make sure that LDL influx by the cells does not lead to Foam cell formation. Cells were incubated for 16 h with LDL/LDL-FITC (although the experiment took only 3 h), watched under a microscope and compared to macrophages incubated for 16 h with oxidized LDL. Morphologically, foam cells have distorted shape, and they are full of oxidized lipid as can be seen in Supplementary Fig. 1C. Supplementary Fig. 1A and B shows that upon LDL/LDL-FITC incubation the cells looked the same as in the control (cells that weren't incubated with LDL/LDL-FITC), what means that no foam cell formation observed upon the LDL/LDL-FITC influx. [file 7124251.f1.docx]
